# Racial Stamina

**Published:** 1899-10-14

**Authors:** 


### The Hospital

A Journal of

The Medical Sciences and Hospital Administration.

Vol. XXVII.—No. 681.

Edited by Sir Henry Burdett, K.C.B., and Solomon C. Smith, M.D., M.R.C.P.

[October 14, 1899.

### Racial Stamina.

Events in South Africa have drawn attention afresh to everything connected with our army, and the fact that a very large proportion of the recruits who offer themselves have to be rejected on account of physical unfitness has led to much lament in regard to the degeneracy of the modern Englishman. The street-bred man is compared, to his serious discredit, with the countryman, and it is held, as, indeed, no one who walks about with his eyes open can avoid seeing, that as man-breeding institutions our cities are a failure. On this hinges the great remedy which is now proposed, a remedy which is expressed in the catch words, "back to the land," or, as some would have it, "back to nature"—as if the life of the agricultural labourer could be called "nature," and as if civilisation had said its last word in regard to the possibility of rearing healthy men in towns.

This is not the place to enter into the politics of the matter, but, when we hear it proclaimed that the cure for the decadence of town-bred man is to be found in schemes of fiscal legislation, having for their aim the protection of agriculture, with the special object of drawing people back upon the land, we feel bound to point out that, effective as such plans might be as a means of providing men of good physique for the defence of the country, they would leave the question of the towns untouched, and that, so far as towns are a cause of degeneracy, they would leave that cause in full activity. If it is true that town life is of necessity destructive to the race—well, we must make up our minds to meet our fate as best we may, and let the civilisation of England, like that of other empires which have gone before it, sink into oblivion. But we do not believe it. We grant that town life as it exists at present is full of evil, and tends to produce a weedy race; but town life as it might be is another matter, and, taking our stand on the possibilities of sanitary science, we feel that the way out of the present difficulty lies in the direction of improving the conditions of our cities rather than in driving people back to the land. It is useless kicking against the pricks; the whole tendency of modern life is townwards, and that form of civilisation will last the longest which best solves the problem of health in towns. That we have not solved it so far is manifest enough, and we have little hesitation in saying that, much as has been done to patch up certain sanitary deficiencies which force themselves upon our attention, no serious attempt has so far been made by the Legislature to root out the main cause of town degeneracy, while the sanitary authorities hold back, almost systematically, from putting in force such powers in this direction as they already possess. Both Parliament, which makes the laws, and sanitary authorities, by whom they are put in force, seem to have adopted it as a broad principle that such overcrowding of great populations on small areas as now exists must not be tampered with. Whenever an improvement is devised, or an insanitary area has to be cleared, it is looked upon as essential that "re-housing" shall be provided in the vicinity for the displaced population; and too often it is the case that, when flagrant examples of insanitation are brought to the notice of the authorities, the latter hold their hands in deference to the plea that the inhabitants of the condemned houses have "nowhere to go." Thus are the evils of overcrowding being nailed fast upon the centres of our great towns, and thus is the present lack of stamina in our town populations being rendered permanent. We are told on every hand that what stands in the way of all improvement in the condition of the masses is rent. But if wages rose along with rent this would not matter; and the reason why wages and rent in the central districts do not rise in equal proportion is that the sanitary authorities, either from lack of will or lack of power, fail to enforce a minimum standard of comfort and of sanitation in their districts. There are always plenty of people who will accept a lower wage and make it out by living, or "pigging," in just those conditions which lead to physical degeneracy, and so long as these people are allowed to pass their lives amid conditions below the accepted sanitary standard it is impossible for decent people to avoid being brought down to the same low level.

It is not by building a few model dwellings here and there, and so crowding still more people on the land; it is not by opening new streets and rehousing the displaced population in lofty "blocks," mortgaging the air of heaven for the sake of finding room; it is not by opening new parks and open spaces in which people can wander only on Bank Holidays; but it is by the unflinching enforcement of a steadily-rising standard of decency and sanitation, among the dwellings of the lowest type, that the conditions of towns will be improved as man-breeding institutions. Rome was not built in a day, and London cannot be unbuilt even in many days; but by a continuous and relentless condemnation of insanitary property, and by a gradual raising of the standard of what is considered "unfit for human habitation," far more would be done towards making London a healthy town than can ever be accomplished by the present insane endeavour to replant the population in the very spots where they have hitherto thriven so ill. Both people and occupations would be driven outwards—and so much the better.

